# Timely immunization completion among children in Vietnam from 2000 to 2011: a multilevel analysis of individual and contextual factors

**DOI:** 10.3402/gha.v9.29189

**Published:** 2016-02-29

**Authors:** Dao Thi Minh An, Jong-Koo Lee, Hoang Van Minh, Nguyen Thi Huyen Trang, Nguyen Thi Thu Huong, You-Seon Nam, Do Van Dung

**Affiliations:** 1 Department of Epidemiology, Institute of Preventive Medicine and Public Health, Hanoi Medical University, Hanoi, Vietnam; 2 JW LEE Center for Global Medicine, Seoul National University College of Medicine, Seoul, Republic of Korea; 3 Department of Family Medicine, Seoul National University College of Medicine, Seoul, Republic of Korea; 4 University of Medicine and Pharmacy at Ho Chi Minh City, Ho Chi Minh City, Vietnam

**Keywords:** timely immunization completion, Vietnam, children under five, MICS

## Abstract

**Background:**

Since the beginning of 2014, there have been nearly 6,000 confirmed measles cases in northern Vietnam. Of these, more than 86% had neither been immunized nor was their vaccination status confirmed.

**Objective:**

To establish the likelihood that children under five in Vietnam had ‘timely immunization completion’ (2000–2011) and identify factors that account for variations in timely immunization completion.

**Design:**

Secondary data from the Multiple Indicator Cluster Survey (MICS), which sampled women aged 15–49 from the 1999 Vietnamese Population and Housing Census frame, were analyzed. Multilevel analysis using Poisson regression was undertaken.

**Results:**

Proportions of children under five who had timely immunization completion were low, especially for HBV dose 2 and HBV dose 3, which decreased between 2000 and 2011. Among seven vaccines used in the National Expanded Program of Immunization (EPI) in 2000, 2006, and 2011, measles dose 1 had the highest timely immunization completion at 65.3%, 66.7%, and 73.6%, respectively, and hepatitis B dose 1 had the lowest at 17.5%, 19.3%, and 45.5%, respectively. Timely immunization completion was less common among children whose mothers had relatively less household wealth, were from ethnic minorities, lived in rural areas, and had less education. At the community level, the child's region of residence was the main predictor of timely immunization completion, and the availability of hospital delivery and community prenatal care in the local community were also determinants.

**Conclusion:**

The EPI should include ‘timely immunization completion’ as a quality indicator. There should also be greater focus and targeting in rural areas, and among women who have relatively low education, belong to minority groups, and have less household wealth. Further research on this topic using multilevel analysis is needed to better understand how these factors interact.

## Introduction

Infectious diseases are major causes of morbidity and mortality in children. One of the most cost-effective and easy ways of contributing to the health and well-being of children is immunization. The goal of immunizing children against illnesses responsible for child mortality and morbidity, such as tuberculosis, polio, diphtheria, pertussis, tetanus, hepatitis B, and measles, is indeed a noble one (1). In May 1974, the World Health Organization (WHO) officially launched a global immunization program, known as the EPI, to protect all children in the world against six vaccine-preventable diseases by the year 2000 (2). The EPI was implemented in Vietnam in 1981. The program was expanded at provincial level throughout the whole country by 1985, covering 100% of districts by 1989 and 100% communes by 1995 (3, 4). With the great efforts of the EPI, Vietnam has achieved excellent success in immunization coverage.

The report by the National Institute of Hygiene and Epidemiology (NIHE) (4) indicated that, from 1993 to 2011, over 90% of children under five in Vietnam received the immunization recommended by the EPI. Vietnam succeeded in eliminating polio in 2000 and dramatically reducing neonatal tetanus by 2005 (4). Despite the impressive efforts of the EPI, in recent years, there continue to be outbreaks of vaccine-preventable diseases in some areas. Since the beginning of 2014, for example, there have been nearly 6,000 confirmed measles cases in the northern areas, of which more than 86% were either not immunized or their vaccination status was not confirmed (5). This low measles vaccine coverage can be attributed in part to adverse events following immunizations (AEFI) using free EPI vaccines (6). Because of concerns about adverse events, some parents held out for foreign vaccines, which they believed were of higher quality. However, these vaccines were always in short supply in Vietnam and this shortage delayed vaccinations (7). In addition, low compliance with ‘timely immunization completion’, as recommended by WHO, may have also been a reason for low vaccination coverage. The WHO report titled *State of Inequality: Reproductive, Maternal, Newborn and Child Health* indicated that both microlevel factors, such as characteristics of children and households, and macrolevel factors, such as the characteristics of health-care systems, policies, geographic and economic factors, contribute to immunization coverage (8).

Timely immunization completion is defined as a child receiving a specific vaccination dose at the point that fell within the schedule recommended by the WHO. The WHO vaccination schedule is as follows (9, 10):

**Table:** 

	Dose (days old)		
	
Vaccine	1	2	3
Bacillus Calmette–Guérin (BCG)	0–28	–	–
Polio	59–88	89–118	119–148
Diphtheria, Tetanus, Pertussis (DPT)	59–88	89–118	119–148
Hepatitis B (Hep B)	29–88	59–118	89–148
Measles-containing vaccines (MCV)	269–328	–	–

For children 12 months and older, 11 doses were recommended. This includes one dose for BCG and measles and three doses for Hep B, DPT, and polio (1, 2, and 3).

Although many studies have measured full immunization coverage, studies on timely immunization completion are rare in developing countries. Thang et al. used data from the Vietnam Demographic and Health Surveys (DHS) to describe improvements in child immunization from 1997 to 2002 and found that children from poor minorities or poor rural backgrounds were the vulnerable groups regarding immunization coverage. However, that study was based on data of children more than 10 years old, and the authors did not include a multilevel analysis (11). The aim of this study was to use the most recently available data in order to establish the likelihood of children under five in Vietnam receiving ‘timely immunization completion’, and identify individual and contextual factors that account for variations in timely immunization completion.

## Methods

### Data source

The global Multiple Indicator Cluster Survey (MICS) program was developed by UNICEF in the 1990s as an international household survey program, to collect internationally comparable data on a wide range of health and social indicators for children and women. The MICS program measures key indicators that allow countries to generate data for use in policies and programs and to monitor progress toward the Millennium Development Goals (MDGs) and other internationally agreed commitments (12). Vietnam was one of the countries that implemented MICS in 1995 (MICS 1), 2000 (MICS 2), 2006 (MICS 3), 2011 (MICS 4), and 2014 (MICS 5) (12–15).

The DHS and MICS contain a vast amount of information on the health and demographic situation of the populations of less developed countries. The primary purpose of the MICS is to assess end-of-decade progress in meeting the goals of the World Summit for Children, whereas the primary purpose of the DHS is to produce indicators for the monitoring and evaluation of population health and nutrition. During the development phase of the end-of-decade MICS questionnaire and manual, UNICEF undertook wide consultation with many organizations, including the DHS development team, in order to achieve comparability across these two survey instruments (16).

This study is a secondary analysis of data from the MICS 2, MICS 3, and MICS 4 conducted in Vietnam. Each of these surveys sampled households and women in the households, aged 15–49 years, based on the 1999 Vietnamese Population and Housing Census framework.

The MICS used two-stage probability stratified and clustered sampling on the Enumeration Areas (EAs), being the smallest unit comprising one or more neighboring blocks. According to the Vietnamese General Statistical Office (GSO), the EAs were fairly homogenous with respect to population, sociodemographic characteristics, economic status, and living conditions. The GSO was responsible for distributing questionnaires to households. Households were sampled from selected EAs by using household lists created from Vietnam's 1999 Population and Housing Census. The MICS 2 and MICS 3 systematically randomly sampled 20 (MICS 2) and one-third (MICS 3) of the households in each EA. The MICS 4 sampled households according to the probability proportional to size method (13–15).

## Measures

### Dependent variables

Three binary dependent variables were derived for this study. The first of these was ‘timely immunization’ for a *specific dose of vaccine*. This was defined according to whether a child received a specific vaccination dose within the schedule proposed by the WHO and recommended by the national EPI. In order to measure this, the ‘time span’ for the given vaccination (date of birth subtracted from date of immunization) was calculated. This was compared with the WHO schedule and the recommendations made by the national EPI (17). Doses were coded as one (correct) if the ‘time span’ fell within the recommended schedule or zero (otherwise).

The second dependent variable was ‘timely immunization completion’ for a *specific vaccine*. This was defined as whether a child received complete recommended doses of a specific vaccine according to his or her age in months, and whether this was ‘timely’, that is, within the recommended timeframe, as explained above. To identify whether a child was vaccinated fully, total numbers of timely vaccination doses were calculated and compared with numbers of vaccination doses recommended by the WHO and the EPI for the child's age in months. The doses were coded as one (fully vaccinated) if the numbers of doses accorded with the recommendations or zero (otherwise).

The third dependent variable was ‘timely immunization completion’ for *all vaccines*, defined as whether the child received all vaccines recommended by the WHO at his or her age in months, and within each vaccine, whether he or she received all the recommended doses, and whether this was ‘timely’, that is, within the recommended timeframe, as explained above. This variable was coded as one, full timely vaccination for all vaccines as recommended, or zero (otherwise). The critical information on dates and specific doses of BCG, polio, DPT, hepatitis B (HBV), and measles vaccinations was collected from immunization cards.

### Independent variables

The independent variables were identified at individual, household, and community levels for the multilevel analysis. The selection of variables was based on evidence of association in general, with correct immunization (18, 19). The single individual-level factor was: 1) the child's birth order, coded as born first or otherwise. Information about the mothers was a proxy for household-level factors. These characteristics were: 1) mother's age, which was grouped as: 15–18, 19–23, 24–28, 29–33, and 34 years and older; 2) marital status (single, married, and divorced); 3) ethnicity, categorized as Kinh or minority; 4) mother's education (below primary, below secondary, below high school, and college or university); and 5) mother's wealth index.

The MICS data set included the Household Wealth Index which was calculated by the GSO of Vietnam. The index was based on the ownership of consumer goods, dwelling characteristics, water and sanitation, and other characteristics related to household wealth. Weights (factor scores) were assigned to correspond with individual household assets (15). The index was divided into quintiles, with the first quintile as the poorest and the fifth as the richest (13). In this study, we used the Household Wealth Index as a proxy for the mother's household wealth.

Information from each EA was used as a proxy for community-level factors. Three variables – ‘community prenatal care by a doctor’, ‘community hospital delivery’, and ‘community mothers’ education’ – were categorized as low or high (using the median as the cutoff). The receipt of prenatal care by a doctor was included as a variable because of evidence that prenatal care can increase mothers’ access to subsequent health-care services including immunization (20). Community hospital delivery was included because the proportion of mothers who deliver in hospital settings can predict child immunization (21). Mothers’ education, defined as the percentage of mothers with secondary or higher education, was included as a factor because higher levels of maternal education are associated with better child health indicators, including a prevalence of child immunization (22).

### Data preparation

Since the MICS 4 in 2011, there has been a reorganization of geographic regions in Vietnam. Eight regions have merged into six. The six regions in this study are: Red River Delta, Northern Midlands and Mountain areas, Northern Central area and Central Coastal area, Central Highlands, South East, and Mekong River Delta. We also combined the mothers’ and children's data sets within each MICS to create one common ‘mother–children’ data set for this analysis.

### Statistical analysis

The sample is described by timely immunization completion. Normalized sampling weights provided by the Vietnamese Population and Housing Census data were used in all analyses to adjust for non-response and produce nationally representative samples.

The proportion of children under five with timely immunization of a *specific dose* was calculated as the number of children who were correctly vaccinated, as per the WHO-recommended schedule for a specific dose, divided by the total number of children vaccinated with the specific dose. The proportion of children with timely and full immunization completion for a *specific vaccine* was calculated as the number of children who were correctly vaccinated as per the WHO-recommended schedule for all doses of a specific vaccine, divided by the total number of children vaccinated with the specific vaccine. The proportion of children under five with timely and full immunization completion for *all vaccines* was calculated by the total number of children with timely vaccination for all doses of all vaccines, as recommended by the WHO schedule at his or her age in months, divided by the total number of children vaccinated with all doses of all vaccines.

A three-level multilevel Poisson regression model was applied in order to account for the hierarchical structure of the DHS data (23). Children (level 1) were nested within households (level 2), which were nested within communities (level 3). Four models were fitted. *Model 1* (empty model) contained no exposure variables. *Model 2* included the individual-level variable (birth order of the children), and *Model 3* included the household-level variables (mother's age, marital status, ethnicity, mother's education, mother's occupation, and mother's Household Wealth Index). *Model 4* included the community-level variables (mother's education, community hospital delivery, community prenatal care by doctor, and mother's region of residence). Incidence rate ratio (IRR) was used as the outcome of the Poisson regression to evaluate determinants at each level.

The random variables A and B have independent Poisson distributions with parameters α_1_ and α_2,_ respectively, and the IRR, using the person-time data, is given by:

$$\text{IRR}=\left(\frac{\text{P}{\text{T}}_{\text{2}}}{\text{P}{\text{T}}_{\text{1}}}\right)\left(\frac{\lambda_{\text{1}}}{\lambda_{\text{2}}}\right)\text{.}$$

In studies with person–time data, the IRR is calculated as the ratio of incidence rates or densities in those exposed to that among those unexposed. An IRR of one indicates that the incidence rates of disease in the exposed and unexposed groups are the same. A value of more than one indicates a positive association or increased risk and a value less than one indicates a negative association or decreased risk among those exposed (24).

MICS data were extracted from SPSS data set supplied by the GSO and converted into Stata 12.0 for cleaning and analysis.

## Results

Table 1 shows the proportions of children under five, who received timely immunization for the 11 specific doses by 2000, 2006, and 2011. In general, the proportions increased between MICS 2 and MICS 4. Measles vaccination had the highest proportion with timely immunization and the first dose of the hepatitis B vaccine had the lowest. For DPT and polio, the proportion of timely immunization completion declined from the first to the third dose in the three surveys, although this was not the case for the HBV vaccine. The second HBV dose had the highest proportion with timely immunization completion. The proportion of timely immunization completion for DPT increased between MICS 2 and MICS 4. This was also the case for polio. In contrast, except for its first dose, timely immunization completion of HBV fell between MICS 2 and MICS 4.

**Table 1:** Proportion of children under five receiving timely immunization by specific doses in Vietnam: 2000, 2006, and 2011

	MICS 2 (*N*=878)		MICS 3 (*N*=2,633)		MICS 4 (*N*=3,520)		
		
MICS vaccines	(*n*)^a^	(%)^b^	(*n*)^a^	(%)^b^	(*n*)^a^	(%)^b^	*p*
BCG	219	43.1	763	49.1	1,396	59.3	<0.001
HBV^1^	214	17.5	743	19.3	1,083	45.5	<0.001
HBV^2^	195	59.2	695	60.2	1,032	52.2	0.01
HBV^3^	177	47.5	628	46.8	841	36.9	0.001
DPT^1^	201	47.2	718	53.1	1,097	64.0	<0.001
DPT^2^	189	43.8	674	44.8	1,056	52.4	0.01
DPT^3^	181	35.5	633	38.7	998	45.4	0.02
Polio^1^	204	50.8	727	52.7	1,258	63.9	<0.001
Polio^2^	191	45.5	684	45.4	1,193	54.5	0.002
Polio^3^	180	42.8	638	40.8	1,111	46.9	0.08
MCV	146	65.3	514	67.7	979	73.6	0.04

*N*=Total of children under five whose immunization cards were showed during the survey.

a Number of children who presented immunization cards during the survey and had written information indicating that he or she was vaccinated for a specific dose.

b Proportion of children who received timely immunization for a specific dose whereby his or her immunization card indicated that he or she was vaccinated for that specific dose.

BCG: Bacillus Calmette–Guérin; HBV: hepatitis B virus; DPT: diphtheria, tetanus, pertussis; MCV: measles-containing vaccines.

Table 2 shows the proportion of timely immunization completion for each specific vaccine by individual, household, and community characteristics. The proportion receiving timely immunization completion was higher for the first born child. For all vaccines except HBV, the proportions receiving timely immunization were higher for children whose mothers were of Kinh ethnicity compared to ethnic minorities, and for children whose mothers had high school or higher education compared with mothers who had lower levels of education. Timely immunization completion was very low for mothers who did not have at least primary education, in particular for polio, DPT, and HBV. Timely immunization completion was higher for currently married mothers, older mothers, and children from households with higher economic status. The wealth gradient was particularly noticeable for the BCG vaccine with 34.1% of the poorest families (first quintile) with timely immunization compared with the 61.2% of the richest families (fifth quintile).

**Table 2:** Proportion of children under five receiving timely immunization completion by specific vaccines in Vietnam from 2000 to 2011

Characteristics	BCG (*n*=2,985)	HBV (*n*=2,146)	DTP (*n*=2,315)	Polio (*n*=2,444)	MCV (*n*=2,064)
Overall	1,496 (50.1)	244 (11.4)	874 (33.8)	954 (34.9)	1,416 (68.6)
Birth order					
1^st^	523 (55.4)	79 (12.4)	299 (41.6)	317 (41.7)	482 (72.9)
Others	973 (47.7)	165 (10.9)	575 (36.0)	637 (37.9)	934 (66.6)
Ethnicity					
Kinh	1,329 (51.8)	207 (11.2)	780 (38.7)	844 (40.0)	1,267 (69.7)
Minority	167 (40.1)	37 (12.6)	94 (31.2)	110 (32.7)	149 (60.8)
Region of residence					
Red River Delta	178 (58.6)	27 (13.0)	90 (43.9)	88 (41.3)	127 (67.2)
Northern Midlands and Mountain areas	166(37.8)	18 (5.9)	127 (37.6)	154 (42.2)	199 (67.2)
North Central area and Central Coastal area	152 (45.8)	32 (14.6)	74 (30.2)	90 (33.7)	154 (72.6)
Central Highlands	439 (42.1)	86 (10.9)	288 (35.0)	309 (35.2)	464 (63.7)
South East	344 (73.7)	43 (12.8)	171 (42.6)	171 (43.3)	275 (72.8)
Mekong River Delta	217 (54.3)	38 (13.1)	124 (40.8)	142 (43.4)	197 (75.5)
Residence					
Urban	563 (59.3)	84 (12.4)	333 (45.2)	360 (46.10	523 (73.9)
Rural	933 (45.8)	160 (10.9)	541 (34.3)	594 (35.7)	893 (65.9)
Education					
Below primary education	506 (48.1)	77 (11.2)	275 (35.4)	325 (37.9)	488 (66.1)
Below secondary education	433 (48.7)	83 (12.1)	268 (36.9)	286 (38.9)	407 (65.5)
Below high school	387 (53.3)	54 (10.5)	213 (39.0)	229 (39.7)	336 (70.0)
College or university	170 (53.6)	30 (11.7)	118 (44.2)	114 (41.8)	185 (82.2)
Marital status					
Currently married	1,458 (50.4)	234 (11.3)	850 (37.9)	925 (39.1)	1,375 (68.6)
Single/divorced	38 (41.7)	10 (14.1)	24 (32.8)	29 (38.7)	41 (69.5)
Age					
15–19	29 (42.7)	2 (5.0)	11 (25.0)	34 (66.7)	22 (68.8)
20–24	250 (50.3)	32 (10.3)	124 (36.5)	230 (63.2)	200 (71.7)
25–34	693 (51.7)	122 (13.1)	406 (49.8)	642 (58.6)	655 (68.8)
≥35	524 (47.4)	88 (10.2)	333 (36.6)	584 (62.5)	539 (67.3)
Mother's Wealth Index					
1st quintile (poorest)	159 (34.1)	39 (12.4)	85 (24.9)	103 (27.3)	166 (56.1)
2nd quintile	215 (42.1)	39 (10.9)	143 (38.3)	156 (38.7)	212 (64.6)
3rd quintile	291 (52.7)	32 (8.4)	145 (33.8)	155 (35.0)	229 (63.4)
4th quintile	336 (51.9)	48 (9.9)	200 (38.8)	227 (42.0)	338 (71.5)
5th quintile (richest)	495 (61.2)	86 (14.1)	301 (45.8)	313 (46.0)	471 (77.7)

*n=*Total number of children in MICS2+MICS3+MICS4, who presented immunization cards during the survey and who had written information indicating that he or she was vaccinated for all doses of a specific vaccine.

Numbers in parenthesis show the proportion of children under five who received timely immunization completion for all doses of a specific vaccine among those who presented immunization cards during the survey and had written information indicating that he/she was vaccinated for all doses of a specific vaccine.

BCG: Bacillus Calmette–Guérin; HBV: hepatitis B virus; DPT: diphtheria, tetanus, pertussis; MCV: measles-containing vaccines.

At the community level, timely immunization completion differed among regions. When comparing regions, for vaccines overall, timely immunization completion was highest for children living in the Mekong River Delta, South East, and Red River Delta regions and lowest in the Northern Midlands and Mountain areas, Northern Central Coastal Areas, and Central Highlands. For all vaccines, timely immunization completion was higher for children in urban rather than rural areas.

Table 3 reports the results from a set of multilevel Poisson regression models. Here timely immunization completion of all vaccine doses was the binary outcome. The IRR of the two levels remained significant in models 2, 3, and 4, respectively, suggesting that household and community-level factors were important for completing timely immunization. The random intercept model with individual-level covariates only (Model 2) provides an indication of the extent to which unobserved characteristics contributed to variations in timely immunization completion.

**Table 3:** Multilevel Poisson regression for determinants of timely immunization completion in Vietnamese children under five from 2000 to 2011

	Model 1	Model 2		Model 3		Model 4	
		
Variables	Coef (95% CI)	Coef (95% CI)	IRR	Coef (95% CI)	IRR	Coef (95% CI)	IRR
**Fixed effects**							
Individual characteristics:							
*Birth order*							
1st born		1		1		1	
Other		0.13 (0.05–0.20)**	**1.14**	0.20 (0.12–0.28)***	**1.22**	0.22 (0.14–0.30)***	**1.24**
Household characteristics:							
*Residence*							
Urban				1		1	
Rural				−0.60 (−0.84 to −0.36)**	**0.55**	−0.31 (−0.56 to −0.07)*	**0.73**
*Mother's education*							
Below primary education				1		1	
Below secondary education				0.22 (0.01–0.42)*	**1.24**	0.19 (−0.03–0.40)**	**1.20**
Below high school				0.27 (0.01–0.53)*	1.31	0.22 (**−**0.04–0.49)	1.25
College or university				0.31 (0.03–0.58)*	**1.36**	0.31 (0.03–0.60)*	**1.37**
*Marital status*							
Currently married				1		1	
Single/divorced				**−**0.098 (**−**0.20–0.07)	**0.91**	−0.11 (−0.21 to −0.002)*	**0.90**
*Mother's age*							
15–19				1		1	
20–24				0.03 (**−**0.27–0.33)	1.03	0.03 (**−**0.27–0.34)	1.04
25–34				**−**0.18 (**−**0.48–0.12)	0.84	**−**0.19 (**−**0.49–0.12)	0.83
≥35				**−**0.24 (**−**0.56–0.08)	0.79	**−**0.30 (**−**0.62–0.02)	0.74
*Mother's Wealth Index*							
1st quintile (poorest)				1		1	
2nd quintile				0.55 (0.25–0.85)***	1.73	0.28 (**−**0.02–0.58)	1.33
3rd quintile				0.63 (0.32–0.94)***	1.88	0.26 (**−**0.06–0.57)	1.29
4th quintile				0.77 (0.45–1.09)***	**2.16**	0.43 (0.11–0.75)**	**1.54**
5th quintile (richest)				1.04 (0.68–1.39)***	**2.82**	0.60 (0.24–0.97)**	**1.83**
*Ethnicity*							
Kinh				1		1	
Minority				−0.26 (−0.46 to −0.06)*	0.77	**−**0.01 (**−**0.25–0.23)	0.99
Community characteristics**:**							
*Community mother's education*							
Low						1	
High						0.01 (0.00–0.01)*	**1.01**
*Community hospital delivery*							
Low						1	
High						0.01 (0.00–0.01)***	**1.01**
*Community prenatal care by doctor*							
Low						1	
High						0.01 (0.01–0.02)***	**1.01**
*Region of residence*							
Red River Delta						1	
Northern Midlands and Mountain areas						**−**0.01 (**−**0.31–0.29)	0.99
North Central area and Central Coastal area						0.40 (0.06–0.74)*	**1.49**
Central Highlands						0.57 (0.22–0.92)**	**1.77**
South East						1.05 (0.69–1.40)***	**2.84**
Mekong River Delta						0.78 (0.43–1.12)***	**2.18**
Random effects	Empty	Child level		Mother level		Community level	
Community level							
Estimate	1.48 (1.35–1.62)	1.49 (1.36–1.63)		1.12 (0.997–1.25)		0.96 (0.85–1.1)	
Variance (SE)	0.07	0.07		0.07		0.06	
MIRR	4.10	4.13		2.90		2.51	
Mother-level							
Estimate	2.05 (1.96–2.14)	2.06 (1.97–2.15)		2.03 (1.93–2.12)		1.99 (1.90–2.09)	
Variance (SE)	0.05	0.05		0.05		0.05	
MIRR	7.06	7.11		6.91		6.68	
Log likelihood	−9,919.3643	−9,913.8514		−9,427.0453		−8,876.6346	
LR test vs. Poisson (*p*)	0.000	0.000		0.000		0.000	

CI: confidence interval; IRR: incidence rate ratio; SE: standard error.

* *p*<0.05

** *p*<0.01

*** *p*<0.001.

Model 3 with both individual and household-level characteristics showed that in addition to children's characteristics, residential area, mother's education, household wealth, and ethnicity were all associated with timely immunization completion among children. Children living in rural areas were less likely to have timely immunization completion (IRR=0.55 with *p*<0.01). Children whose mothers had secondary education, high school, or college/university education were significantly more likely to have had timely immunization completion compared with mothers who received only primary educations (IRR 1.31 and 1.36, respectively, *p*<0.05). The higher the family's household wealth, the greater the likelihood of the child receiving the correct number of immunizations (IRR: 1.73, 1.88, 2.16, and 2.82, respectively with *p*<0.001). Children from minority ethnic groups were less likely to receive timely immunization completion compared with children of Kinh ethnicity (IRR=0.77 (*p*<0.05)).

Model 4 included individual and household characteristics, and also community-level factors. In addition to the five determinants identified in models 2 and 3, there were other factors associated with children's timely immunization completion. Children from highly educated communities with better health services (measured by hospital deliveries and prenatal care by a doctor) were more likely to have had timely immunization completion. With the exception of children living in the Northern Midlands and Mountain areas, children living in all other regions were more likely to have had timely immunization completion compared with children living in the Red River Delta.

## Discussion

The findings show that the proportion of children who received timely immunization completion for DPT, polio, MCV, BCG, and HBV dose 1 increased gradually from 2000 to 2011. These achievements may be in part explained by the efforts of the EPI especially with support from the Global Alliance for Vaccines and Immunization (GAVI) since 2003 (4, 13–15). However, this study indicated that ‘timely immunization’ for a specific dose among children under five between 2000 and 2011 was low (Table 1), especially for HBV vaccine dose 2 and dose 3. Up until now, the main indicator used by the EPI in Vietnam to measure the success of the program was the percentage of full immunization coverage. Indicators of timely immunization completion have not been included as official indicators (26).

Low timely immunization was shown in a study conducted by the DHS within the last 10 years in 31 countries. The DHS highlighted that the invalid vaccinations (diphtheria, tetanus, pertussis (DTP^1^, DTP^3^)) and MCV across all countries were 12.1% (inter quartile range, 9.4–15.2%), 5.7% (5.0–7.6%), and 15.5% (10.0–18.1%), respectively. Of the invalid DTP^1^ vaccinations, 7.4 and 5.5% were administered to children less than 1 or 2 weeks of age, respectively. In 12 countries, the proportion of invalid DTP^3^ vaccinations administered with an interval of less than 2 weeks before the preceding dose, varied between 30 and 50%. In 13 countries, the proportion of MCV doses administered to children less than 6 months of age varied between 20 and 45% (27). According to the WHO's recommendations, timely immunization is very important in enhancing efficacy and effectiveness of immunization. The Vietnamese EPI should seriously consider using timely immunization completion as a quality indicator in addition to full vaccine coverage.

The current study shows a low prevalence of timely immunization completion among Vietnamese children under five. One of the reasons for delayed childhood vaccination could be that parents and health workers were afraid of possible adverse events following immunization (AEFI). The report of the 19th meeting of the Technical Advisory Group on Immunization and Vaccine-Preventable Diseases in the Western Pacific Region (August, 2010) indicated that there were 12 severe cases with AEFI reported in 2007–2008. It has been suggested that this was a reason for the dramatic decline in the coverage for both routine hepatitis B vaccination and birth dose in 2007 (67% and 27%, respectively) (28). There was also a huge measles outbreak in the northern area of Vietnam between late 2013 and early 2014. During this outbreak, there were 9,474 confirmed measles cases of which 81.1% were identified as not receiving immunization (29). This shows the consequences of low immunization coverage. Therefore, in order to improve timely immunization completion in Vietnam, the EPI should take into account that parents and health workers might be reluctant to support vaccination because of local knowledge about AEFI. For example, in 2013 there were 43 serious cases of AEFI reported for the Quinvaxem vaccine in Vietnam which had an impact on public perception (6). Nonetheless well-informed education should be given to both parents and health workers about the benefits of legitimate and timely immunization completion, especially where there are reports of AEFI.

This study shows that the factors associated with timely immunization completion include individuals, households, and community-level factors. At the individual level, a child who was not first born was more likely to have timely immunization completion doses than a first born child (Table 3). However, a study in Nigeria indicated that there were no differences between the first born and other children in regard to full immunization (21). In contrast, a multilevel study in India indicated that children who were not born first are 16% less likely to be fully immunized than the first child (30). Similar findings have been reported in Bangladesh where second born and subsequent children were less likely that the first born child to receive full immunization (31).

At the household level, this study clearly depicts that the child's residential area, mother's education, household wealth, and mother's ethnicity were all significantly associated with timely immunization completion in both univariate and multilevel analysis (Tables 2 and 3). Other studies in India, Pakistan, Nigeria, Bangladesh, and Sub-Saharan Africa showed that children whose mothers were of lower education or lived in rural areas were less likely to receive full immunizations (21, 22, 32, 33). A 2013 study conducted in China, a country that borders Vietnam, also showed that the proportion of children receiving full immunization was almost two and a half times higher for children whose mothers graduated from high school compared with children whose mothers’ highest level of education was primary school or less (18).

The current study also shows that household wealth is an important factor. Similarly, studies in Nigeria and Sub-Saharan Africa indicated that household socioeconomic status was associated with health-related outcomes, such as health-seeking behaviors, the utilization of primary preventive services, and vaccination (21, 33). We would expect that women with greater household wealth can more easily afford to provide better health-care services for their children (34). A study in 31 countries in the WHO African Region indicated that DTP^3^ immunization coverage was lower in children from poorer areas. Among the studied countries, DTP^3^ immunization coverage increased with rising economic status. Half of the countries achieved coverage of at least 73% in the poorest wealth quintile, whereas half of the countries in the richest wealth quintile reported coverage of over 86% (27).

Our study also indicates that children of Kinh ethnicity were more likely to receive timely immunization completion compared with children from minority ethnic groups. Different ethnicity and residential areas in Vietnam not only reflect different attitudes and health-seeking behaviors but also disparities in socioeconomic positions. The gap between the rich and the poor, between Kinh and other ethnic minority groups, between urban and rural areas are concerning issues in Vietnam. The Kinh who live mainly in the plains, near the rivers, and in urban areas, are more likely to benefit from better socioeconomic conditions. On the other hand, most ethnic minority groups are living in the highlands and rural and mountainous areas. People in these areas generally have relatively poorer socioeconomic conditions. The poverty rate in Vietnam's minority ethnic groups in 2008 was 49.8% compared with 8.5% for people of Kinh ethnicity. In 2006, 40% of poor children lived in rural areas, while only 10% lived in the cities (35). Similar findings have been reported in studies in Nigeria, which show that children of mothers’ from the Igbo ethnic group (which is the main ethnic group in Nigeria) had more than twice the likelihood of receiving full immunization compared with children of Hausa/Fulani/Kanuri mothers (ethnic minority) (21).

At the community level, Model 4 (Table 3) indicates that although mothers’ education and community health-care services had little influence on timely immunization, community factors did indeed contribute to timely immunization completion. Similar results are seen in studies conducted in 31 countries in the African region. They found that community home delivery – as a proxy of health-care system – was not associated with full immunization (27). In contrast, a study in Ethiopia showed that community-level factors were associated with the number of childhood immunizations, net of individual-level covariates. Children who lived in communities with high average education had almost 20% more immunizations compared with children from communities with low education (36). A study in Malawi found that regions with a high percentage of deliveries attended by health personnel were also characterized by a higher coverage of vaccination (37).

The region of residence was the most significant factor associated with timely immunization completion. In this study children living in the Northern Midlands and Mountain areas were less likely to receive timely immunization completion than those living in other regions. However, these areas border two provinces in Southern China. Transportation is difficult and the population is largely comprised of ethnic minorities. A study in Nigeria indicated that children living in the South East and Southern regions in Nigeria, characterized by extensive mangrove forests, lagoons and swamps stretching over hundreds of kilometers inland, as well as poverty, poor social infrastructure and conflicts, were less likely to have completed timely immunization (21).

### Strengths and limitations

There are some limitations to this study. Firstly, the estimates were derived only from available information on vaccination cards and children were excluded from this study if they did not have an immunization card. Mothers who reported immunization in their MICS interviews but did not have an immunization card were not included. Secondly, although we attempted to separate the determinants of timely immunization completion into hierarchical levels, this study used secondary data collected from the MICS that was not intended for multilevel analyses. Hence proxy measures were used (e.g. mothers’ education, prenatal care, and hospital deliveries). However, this study was the first of its kind in Vietnam. It is important for at least two reasons. First, because it measured timely immunization completion when most other studies have looked at immunization coverage in general, and second, because it conducted a multilevel analysis to look at both individual and contextual factors associated with timely immunization.

## Conclusions

The EPI in Vietnam should focus more on mothers in rural areas who have lower education, belong to minority ethnic groups, and are poor. It should also take into account timely immunization completion as a quality indicator for assessment and evaluation of vaccination programs. The EPI should educate the community regarding the importance of timely immunization completion and ensure that data collected in the future allow for rigorous multilevel analysis to better inform policy on childhood immunization.
